# The New Serum-Free OptiPASS^®^ Medium in Cold and Oxygen-Free Conditions: An Innovative Conservation Method for the Preservation of MDA-MB-231 Triple Negative Breast Cancer Spheroids

**DOI:** 10.3390/cancers13081945

**Published:** 2021-04-18

**Authors:** Antoine Goisnard, Clémence Dubois, Pierre Daumar, Corinne Aubel, Marie Depresle, Jean Gauthier, Bernard Vidalinc, Frédérique Penault-Llorca, Emmanuelle Mounetou, Mahchid Bamdad

**Affiliations:** 1 Imagerie Moléculaire et Stratégies Théranostiques, Institut Universitaire de Technologie, Université Clermont Auvergne, UMR INSERM-UCA, U1240, 63000 Clermont Ferrand, France; antoine.goisnard@uca.fr (A.G.); clemence.dubois@uca.fr (C.D.); pierre.daumar@uca.fr (P.D.); emmanuelle.mounetou@inserm.fr (E.M.); 2 BIOMARQUEURS Company, 5 Avenue Blaise Pascal, 63178 Aubière, France; marie.depresle@uca.fr (M.D.); jean.gauthier@uca.fr (J.G.); bernard.vidalinc@uca.fr (B.V.); 3 Imagerie Moléculaire et Stratégies Théranostiques, Faculté de Médecine, Université Clermont Auvergne, UMR INSERM-UCA, U1240, 63000 Clermont Ferrand, France; corinne.aubel@uca.fr; 4 BIOPASS Company, 5 Avenue Blaise Pascal, 63178 Aubière, France; 5 Imagerie Moléculaire et Stratégies Théranostiques, Centre de Lutte Contre le Cancer Jean Perrin, Université Clermont Auvergne, UMR INSERM-UCA, U1240, 63000 Clermont Ferrand, France; frederique.penault-llorca@clermont.unicancer.fr

**Keywords:** triple-negative breast cancer, preclinical spheroid models, MDA-MB-231 spheroids, new spheroid storage concept, drug screening

## Abstract

**Simple Summary:**

Cancer spheroids are reproducible and relevant multicellular in vitro preclinical models. Thus, their use is required more and more for drug development processes in oncology in order to improve the prediction of anticancer drugs responses. Moreover, spheroid models allow for the reduction in animal experimentation, in accordance with the rule of Reduce, Refine, Replace (3Rs). In order to optimize and extend the use of these spheroid models, this works was focused on the development of an original methodology to keep these cancer spheroids in the long term. This innovative concept is based on a cold storage for up to 7 days of Triple-Negative Breast Cancer (TNBC) spheroids cultured in the synthetic serum-free OptiPASS^®^ culture medium. Major spheroid characteristics could be preserved with this new conservation method, allowing their use in high throughput screening tests.

**Abstract:**

Cancer spheroids are very effective preclinical models to improve anticancer drug screening. In order to optimize and extend the use of spheroid models, these works were focused on the development of a new storage concept to maintain these models in the longer term using the Triple-Negative Breast Cancer MDA-MB-231 spheroid models. The results highlight that the combination of a temperature of 4 °C and oxygen-free conditions allowed the spheroid characteristics of OptiPASS^®^ serum-free culture medium to preserve the spheroid characteristics during 3-, 5- or 7-day-long storage. Indeed, after storage they were returned to normal culture conditions, with recovered spheroids presenting similar growth rates (recovery = 96.2%), viability (Live/Dead^®^ profiles) and metabolic activities (recovery = 90.4%) compared to nonstored control spheroids. Likewise, both recovered spheroids (after storage) and nonstored controls presented the same response profiles as two conventional drugs, i.e., epirubicin and cisplatin, and two anti-PARP1 targeted drugs—i.e., olaparib and veliparib. This new original storage concept seems to induce a temporary stop in spheroid growth while maintaining their principal characteristics for further use. In this way, this innovative and simple storage concept may instigate future biological sample preservation strategies.

## 1. Introduction

Cancer remains a global health care issue, with 18.1 million new diagnosed cases and 9.6 million deaths worldwide in 2018 [1]. Particularly, the Triple-Negative Breast Cancer (TNBC) subtype is characterized by an absence of estrogen and progesterone receptor expression and the absence of epidermal growth factor receptor-2 (HER2) overexpression [2]. This very heterogeneous pathology has a poor prognosis due to a high aggressiveness and a frequent rate of local and metastatic recurrences [3]. Recently, precision medicine has introduced targeted therapies to improve the treatment of these tumors, such as the Poly ADP-Ribose Polymerase (PARP) inhibitors family [4]. However, only 30% of TNBCs present a BRCAness phenotype, generating heterogeneity in the sensitivity of PARP inhibitors [5]. This is why drug discovery studies need to be carried out with the objective of TNBC treatment optimization [6], implying explorations of preclinical models. One major limitation in the pharmaceutical research field is the significant failure rate of anticancer drugs validation during clinical trials [7,8]. To improve the oncological drug development process and reduce time and costs spent in inconclusive trials, it is essential to increase the effectiveness and the prediction of the drug response in patients [9]. This is why, during the last decade, efforts have been made to develop preclinical predictive models in order to improve cancer development studies and anticancer treatments, especially for aggressive tumoral subtypes [8].

In this context, three-dimensional (3D) cell cultures models appeared as a pertinent in vitro option. More precisely, multicellular spheroids are compact round cell aggregates generated from cancer cell lines [10,11]. Their principal asset is to reproduce important cell–cell and cell–extracellular matrix interactions in the avascular tumorigenesis phase. Indeed, these interactions are major elements of the cancer microenvironment, indispensable for the maintenance of intercellular communications and mechanical forces [12,13]. Spheroid compaction is also at the origin of an avascular tumor-like cell organization with the generation of nutrient, oxygen, pH and metabolic waste removal gradients. Tumoral cells thus form different metabolic layers with a hypoxic core enriched with necrotic cells, middle layers of quiescent cells and a proliferative front at the periphery. All these characteristics allow cell metabolic heterogeneity and a gene expression profile close to those observed in in vivo avascular solid tumors to be recreated [14,15]. Thereby, in the drug development process, spheroid models are filling the gap between the proof of concept made for classic monolayer cell cultures and the preclinical final validation for animal models [16]. Indeed, they more precisely recapitulate in vivo solid tumor characteristics than classic cell cultures and discard some animal experimentation limitations linked with experiment number, costs and ethical issues [17,18].

The predictability and reproducibility of the 3D culture models’ drug responses depend on various parameters, such as the composition of the culture medium [19,20]. The majority of cell culture media are supplemented with fetal calf serum (FCS), which presents several disadvantages in terms of reproducibility [21,22]. The use of a chemically defined serum-free synthetic media in cell culture systems appears now to be a key element for reproducibility of in vitro models. In our previous works, two spheroid models obtained from two TNBC cell lines, MDA-MB-231 and SUM1315, were developed in the serum-free OptiPASS^®^ medium [23]. These works presented clearly optimized spheroid growth and viability for OptiPASS^®^ cultured models compared to others cultured in the serum-supplemented medium. Moreover, OptiPASS^®^ medium improved the spheroids’ drug sensibility threshold and also allowed a prolonged culture of spheroids. Alongside this, other studies recently highlighted an innovative approach to store colorectal multicellular tumor spheroids under cold (4 °C) and anoxic conditions [24]. This methodology allowed spheroid integrity to be maintained for 18 days without changing the response to anticancer treatment.

In the context of the development and optimization of predictive preclinical models in oncology, this work aimed to evaluate the potential of using the synthetic OptiPASS^®^ medium in combination with anoxia and cold storage conditions, for the preservation of TNBC MDA-MB-231 spheroids, over time.

## 2. Materials and Methods

### 2.1. Maintenance of Cell Cultures

The triple-negative breast cancer MDA-MB-231 cell line (ATCC^®^, HTB26™) was stored in the Biological Resource Center of Jean Perrin Comprehensive Cancer Center under the No. BB-0033-00075 (Clermont-Ferrand, France). After absence of mycoplasma contamination check (Mycoplasmacheck test, Eurofins Genomics, Luxembourg), MDA-MB-231 cells were cultured in RPMI 1640 medium (Gibco, Dublin, Ireland) supplemented with 10% decomplemented fetal calf serum (Eurobio Scientific, Paris, France) and 20 µg/mL Gentamycin (Panpharma, Paris, France) at 37 °C under 5% CO_2_ in humid incubator.

### 2.2. Spheroids Formation, Culture and Storage

MDA-MB-231 spheroids were formed as previously described [23,25]. Briefly, cells in suspension in RPMI 1640 (Gibco) or OptiPASS^®^ (BIOPASS Society, Aubière, France) were seeded in ultralow attachment round-bottom microplates (Corning, New-York, NY, USA, catalogue #4520) at a concentration of 1000 cells/well. After 24 h at 37 °C in humid incubator, Geltrex LDEV-Free Reduced Growth Factor Basement Membrane Matrix (Gibco, Dublin, Ireland, catalogue #A1413202) diluted in cold RPMI1640 or cold OptiPASS^®^ culture medium was dispensed in each well for a final concentration of 2%. Microplates were then shacked for 20 min at 200rpm to promote the formation of a unique cell aggregate per well. The day after, compact spheroids were observable in bright field microscopy.

For both conditions, spheroid size and integrity were measured by bright field microscopy at days D1, D3, D4, D7, D10 and D14. Spheroid viability was assessed at D3, D4, D7 and D14 using Live/Dead^®^ fluorescence microscopy test. Metabolism activity of spheroids was also quantified at D3, D7 and D14 with resazurin metabolic activity test.

**Figure 1:**
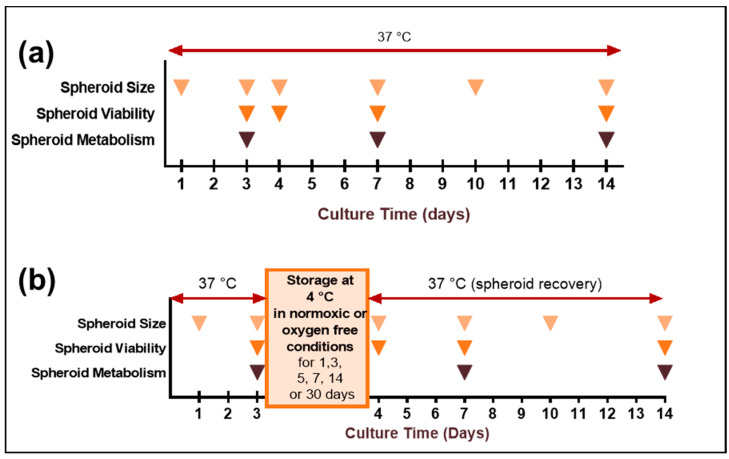
Schematic representation of MDA-MB-231 spheroids cold storage experiments. MDA-MB-231 spheroids were cultured in RPMI 1640 fetal calf serum-supplemented or OptiPASS^®^ medium and maintained in classic cell culture conditions (humid incubator, 37 °C, 5% CO_2_). (**a**) For nonstored control condition, spheroids were continuously maintained at 37 °C, i.e., in classic culture conditions, for 14 days. (**b**) For storage experiments, after 3 days of culture, the spheroids in microplates were stored at 4 °C in normoxic or oxygen-free conditions for 1, 3, 5, 7, 14 or 30 days. After storage, spheroids in microplates were replaced in classic culture conditions for 10 supplemental days—i.e., between the 4th and the 14th days of culture.

After 3 days of culture, microplates were stored for different times at 4 °C or remained in the incubator in normal culture conditions (control). For the oxygen-free storage condition, microplates were packed in a hermetically closed plastic bag (Flo™, Marcilly, France, catalogue #763783) with anaerobic gas generating AnaeroGen™ 2.5L (Oxoid™, Dardilly, France, catalogue #AG0025A) before being kept at 4 °C for storage period of 1, 3, 5, 7, 14 or 30 days (Figure 1). The confirmation of the anaerobic atmosphere (0% O_2_) was checked with monogaz O2 detector (Honeywell, Charlotte, NC, USA) (Appendix A) and Resazurin Anaerobic Indicator (Oxoid™, Dardilly, France, catalogue #BR0055B). After each storage time, microplates were taken out of the bags and 20 µL of RPMI1640 or OptiPASS^®^ culture medium was added to each well, before replacing microplates in normal cell culture conditions (37 °C, 5% CO_2_ in humid incubator) for 11 other days, giving a total culture time of 14 days (Figure 1). For control nonstored condition, microplates were maintained continuously for 14 days in normal culture conditions with a 20 µL RPMI1640 or OptiPASS^®^ culture medium addition per well at the 4th culture day.

### 2.3. Spheroids Growth Evaluation

Each spheroid of the microplates was automatically imaged using bright field microscopy module of the Cytation™3MV cell analyzer (Biotek^®^-M = 4X) coupled with Gen5 3.08 software (Biotek^®^, Winooski, VT, USA). The “Cellular analysis” tool was applied to a stitched picture to calculate the object size of each well (threshold = 20,000, background = light, min. object size = 150 µm, max. object size = 1000 µm). Spheroid size was recorded for each condition on the 1st, 3rd, 4th, 7th, 10th, and 14th days of culture (D1, D3, D4, D7, D10 and D14) allowing growth kinetic curves to be set. Growth curve slopes were calculated for each spheroid condition between the 4th and the 14th culture days (corresponding to the poststorage period) [26].

### 2.4. Spheroids Viability Assessment

The spheroid cell viability was monitored after 3, 4, 7 and 14 days of culture (D3, D4, D7 and D14) using Live/Dead^®^ fluorescent microscopy kit (Invitrogen, Carlsbad, CA, USA, catalogue #L3224). Spheroids were incubated for 30 min at room temperature in a working solution containing 1 µM calcein-AM and 2 µM ethd-D1 in D-PBS (Sigma, Saint-louis, MI, USA, catalogue #D8537). They were then transferred to a µ-Slide 8 Well (Ibidi, catalogue #µ-Slide 8 Well) to be imaged using the fluorescent microscopy module of the Cytation™3MV cell analyzer (Biotek^®^-M = 4X-fluorescence filters = green fluorescent protein and propidium iodide).

### 2.5. Spheroid Metabolic Activity Assay

Additionally, global spheroid metabolic activity was measured at days 3, 7 and 14 (D3, D7 and D14) by transferring each spheroid to 100 µL of a sterile 60 µM resazurin solution in D-PBS (ACROS Organics™, Waltham, MA, USA, catalogue # 189900010). This test was based on the transformation of resazurin to resorufin by the mitochondrial respiration chain in living cells. The resorufin concentration, which can be measurable by fluorimetry, was directly proportional to the metabolic activity in spheroids [27]. After 17 h incubation at 37 °C, the fluorescence intensity of resorufin (593 nm) was measured using Cytation™3MV fluorimetry module (gain = 70). Blank subtraction was applied to data obtained from spheroid-free wells.

### 2.6. Spheroid Tumoral Proliferation Gradient Analysis

Spheroids cultured in OptiPASS^®^ medium without storage (control spheroids) or stored for 3, 5 or 7 days at 4 °C in oxygen-free conditions were harvested at days 4, 7 and 14.

For ki67 immunostaining, after a 12 h fixation in paraformaldehyde 4% (Sigma-Aldrich catalogue #P6148), spheroids were successively incubated in 15 and 30% sucrose D-PBS solutions to be dehydrated. Spheroids were then placed in cryostat embedding medium to be quickly frozen in liquid nitrogen and cut into 10 µm sections with a cryostat device (CICS, Université Clermont Auvergne, France). Obtained slides were then incubated in saturation/permeabilization solution (PBS, 1%BSA, 0.5X Triton) for 1 h. They were then incubated with PBS 0.1% BSA diluted primary antibodies anti-ki67 (polyclonal rabbit, 1/250, Merck, Darmstadt, Germany, catalogue #AB9260), or Mouse IgG1 isotypic control for 1 h. After three washes in PBS, slides were incubated with PBS 0.1% BSA diluted secondary fluorescent antibodies antirabbit (Donkey anti-Rabbit IgG coupled to Alexa Fluor™568, 1/800, Invitrogen, Carlsbad, CA, USA, catalogue # A10042) for 1 h, followed by a nuclear counterstaining with Hoechst33258 (Sigma, Saint-Louis, MI, USA, catalogue #B2883; 1 µg/mL) for 5 min. Negative controls with no primary antibodies were also performed. Slides were mounted with Prolong Diamond Antifade (Invitrogen, Carlsbad, CA, USA, catalogue # P36970) and imaged with Cytation™3MV fluorescent microscopy module (M = 10X-fluorescence filters = Texas Red and DAPI). Thanks to the “cellular analysis” algorithm of Gen5 3.08 software, nuclei were detected and localized on DAPI channel (threshold = 5000, background = dark, min. object size = 5 µm, max. object size = 50 µm). For ki67 staining, some nuclei presented intense and specific staining compared to other ones, agreeing with an on/off activation of the transcription factor expression depending on cell cycle. Thereby, adapted algorithm was used to select nuclei subpopulation presenting activated ki67 expression (Texas Red fluorescent intensity > 3000). For the quantification of ki67 expression in each spheroid, the following formula was applied:

(1) $$\begin{array}{c} Global\;marker\;expression\;in\;the\;spheroid\\ =\%\;ki67positive\;nuclei\;per\;spheroid\times mean\;fluorescent\;intensity\;of\;positive\;nuclei \end{array}$$

### 2.7. Spheroid Hypoxia Level Analysis

For spheroid global hypoxia level evaluation, ROS-ID^®^ orange reagent (EnzoLifeSciences, Farmingdale, NY, USA, catalogue #ENZ-51042) was used. This probe was attached to a nitro group that was specifically reduced by nitro reductase activity in hypoxic cells, releasing an orange fluorescence. Spheroids were exposed with Hypoxia Red reagent at a concentration of 250 µM diluted in OptiPASS^®^ medium for 4 h in humid incubator at 37 °C and 5% CO_2_ (Figure S1). They were then transferred to a µ-Slide 8 Well (Ibidi, Gräfelfing, Germany, catalogue #µ-Slide 8 Well) to be imaged using the fluorescent microscopy module of the Cytation™3MV cell analyzer (Biotek^®^, Winooski, VT, USA, M = 4X, fluorescence filter = Texas Red). Mean fluorescence intensity of spheroids was calculated with “cellular analysis” algorithm of Gen5 3.08 software (threshold = 9000, background = dark, min. object size = 100 µm, max. object size = 1000 µm).

### 2.8. Anticancer Drugs Solubilization and Exposure 

Epirubicin (Carbosynth, Compton, UK, catalogue #FE22741) and cisplatin (Carbosynth, Compton, UK, catalogue #FC20460) were solubilized in distilled water and DMSO, respectively, to prepare 10 mM stock solution. Adapted dilutions were prepared in OptiPASS^®^ medium for final treatment concentrations of 0.1, 1 or 10 µM. Olaparib (Carbosynth Compton, UK, catalogue #FO33122) and veliparib (BOC sciences^®^, Ney York, NY, USA, catalogue #912445-05-7) were solubilized in DMSO to prepare 50 mM stock solution. Adapted dilutions were prepared in OptiPASS^®^ medium for final treatment concentrations of 0.5, 5 or 50 µM. With the exception of epirubicin treated samples, the final DMSO concentration remained constant at 0.1% in all treated spheroid conditions.

### 2.9. Spheroids Anticancer Drug Treatments 

MDA-MB-231 spheroids cultured in OptiPASS^®^ medium without the storage step or stored for 3, 5 or 7 days at 4 °C in oxygen-free conditions were treated for 5 days (between the 5th and the 10th culture days), with increased concentrations of selected anticancer drugs. The size of the untreated control and treated spheroids was monitored daily using Cytation™5MV (Biotek^®^, Winooski, VT, USA) coupled with BioSpa™ automated cell incubator (Biotek^®^, Winooski, VT, USA) and Gen5 3.08 software (Biotek^®^, Winooski, VT, USA). Growth curve slopes were calculated for each spheroid between the 5th and the 10th culture days. At the end of the treatment, spheroid viability was assessed by the Live/Dead^®^ and resazurin tests as described previously. Normalized viability was determined by the percentage of treated spheroids resorufin values on untreated control values.

### 2.10. Statistical Analysis

Results were presented as mean ± standard deviation. All experiments were performed independently on at least three separate occasions. An unpaired 2-sided Student’s *t*-test or two-way ANOVA (confidence interval = 95%, Dunnett’s multiple comparison) was used to evaluate statistical significance. Results were considered statistically different when *p* < 0.05 (*). Stronger differences were noted as follows: *p* < 0.01 (**), *p* < 0.001 (***), *p* < 0.0001 (****) and *p* < 0.00001 (*****). Nonsignificant results were noted as “ns”.

## 3. Results

### 3.1. Normoxic and Cold Storage Action on MDA-MB-231 Spheroid Preservation in RPMI1640 and OptiPASS^®^ Culture Media

MDA-MB-231 spheroid models cultured for three days in RPMI1640 or OptiPASS^®^ medium were firstly stored in normoxic conditions at 4 °C, for one to three days. Then, 3D cell cultures were then exposed to standard cell culture conditions—i.e., at 37 °C under 5% CO_2_ [23]. Recovery was analyzed for 11 days using several cell culture parameters such as spheroid integrity, spheroid growth, cell viability/mortality and cell metabolic activity in comparison to nonstored control spheroids cultured in RPMI 1640 medium or OptiPASS^®^ medium, respectively.

**Figure 2:**
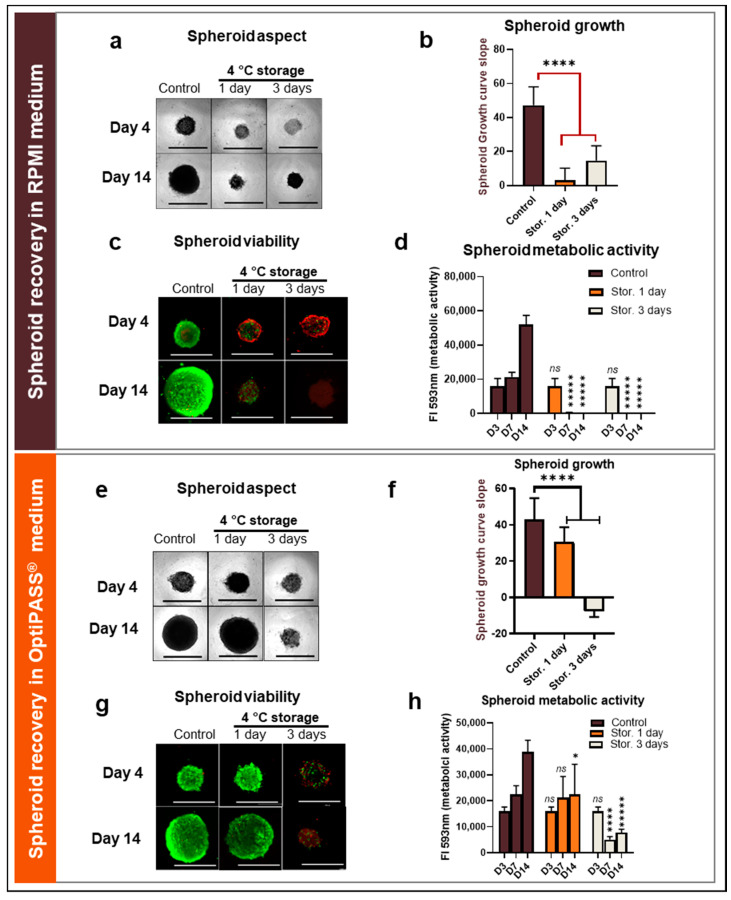
MDA-MB-231 spheroid preservation analysis after 4 °C storage in RPMI1640 and OptiPASS^®^ media culture. Three-day-old spheroids were placed for 1 or 3 days at 4 °C before being exposed to normal culture conditions—i.e., 37 °C, 5% CO_2_ in both RPMI1640 and OptiPASS^®^ medium cultures. Spheroid integrity aspect and size (**a**) in RPMI and (**e**) in OptiPASS^®^ were analyzed with bright field microscopy and object size algorithm (Cytation™3MV, Gen5, BioTek^®^-M = 4X, scale bar = 1000 µm). Spheroid cell proliferation was measured by the analysis of growth curve slopes between D4 and D14 (**b**) in RPMI and (**f**) in OptiPASS^®^, reflecting proliferation capacities and recovery of cell spheroids after storage step. Spheroid cell viability after storage was analyzed by Live/Dead^®^ tests (**c**) in RPMI and (**g**) in OptiPASS^®^ with green fluorescence for viable cells and red fluorescence for dead cells, using Cytation™3MV equipped with GFP and IP fluorescence cubes (M = 4X, scale bar = 1000 µm). Spheroid cell metabolic activity change after storage was quantified using the resazurin test (**d**) in RPMI and (**h**) in OptiPASS^®^, at D3, D7 and D14 with normalized 593 nm Fluorescence Intensity (FI) measures. For each storage condition, significances compared to nonstored condition were indicated as ns (not significant; *p* > 0.05), * *p* < 0.05, **** *p* < 0.0001, ***** *p* < 0.00001.

In RPMI 1640 culture medium, the integrity, compaction level and size of spheroids were followed over time. For spheroids stored for one or three days, a translucent and less cohesive aspect was detected at day 4, in comparison to nonstored control spheroids (Figure 2a). In contrast, after 14 days of culture, the spheroids previously stored for one and three days at 4 °C presented the same aspects as nonstored controls (Figure 2a). However, at D14 spheroid size after one day (374.3 ± 66.7 µm) and three days (478.2 ± 37.1 µm) of 4 °C storage remained greatly lower compared to nonstored control cells at 890.9 ± 45.4 µm (*p* = 10^−113^ and 10^−86^, 2-sided *t*-test). Then, spheroid cell proliferation was analyzed between the 4th and the 14th days of culture by a growth curve slope analysis. Slopes of nonstored control spheroids were 47.3 ± 10.9 and clearly superior to one- or three-day storage conditions, at 3.0 ± 7.2 (*p* = 10^−72^, 2-sided *t*-test) and 14.7 ± 8.6 (*p* = 10^−37^, 2-sided *t*-test), respectively (Figure 2b). The viability analysis of poststorage spheroids, using the Live/Dead^®^ fluorescent imaging kit, showed a large part of red dead cells located in the periphery of the spheroids for both storage conditions at day 4 (Figure 2c). Similarly, at the 14^th^ day of culture, only a poor or absent viable green cells population remained in spheroids. In contrast, nonstored control spheroids presented cohesive viable green cell masses at D4 and D14 (Figure 2c). Finally, the metabolic activity of spheroids was monitored for each storage condition at D3, D7 and D14 using the resazurin test. For nonstored control spheroids, the metabolic activity increased regularly over time, with 16.1 ± 4.5 × 10^3^ UF (fluorescence intensity units) at D3 to 21.4 ± 2.8 × 10^3^ UF at D7 and to 52.0 ± 5.5 × 10^3^ UF at D14 (Figure 2d). In contrast, after one and three days of cold storage, the metabolic activity of spheroids decreased dramatically at D7 (0.3 ± 0.5 × 10^3^ UF) and D14 (0.2 ± 0.1 × 10^3^ UF) (*p* = 10^−3^, 2-sided *t*-test) compared to 16.1 ± 4.5 × 10^3^ UF at D3 (before storage) (Figure 2d).

In OptiPASS^®^ culture medium, after one day of cold storage, the spheroid compaction aspect was similar to controls at day 4 and day 14 (Figure 2e). Interestingly, for this condition, spheroid size was close to those of nonstored controls at day 14 (816.1 ± 58.8 µm compared to 908 ± 52.1 µm; *p* = 10^−15^, 2-sided *t*-test) (Figure 2e). In contrast, after three days of cold storage in OptiPASS^®^ medium, spheroids were less compact and significantly smaller than nonstored spheroid controls at day 14 (483.4 ± 34.6 µm compared to 908 ± 52.1 µm; *p* = 10^−108^, 2-sided *t*-test) (Figure 2e). For growth curve slope parameter analysis, after one day of spheroid storage in OptiPASS^®^ medium, the slopes were significantly lower (30.5 ± 8.3) than controls (43.1 ± 11.6) (*p* = 10^−17^, 2-sided *t*-test) (Figure 2f). For spheroids stored for three days, the growth curve slopes were negative with −7.6 ± 3.1 and clearly lower to those of nonstored spheroids (*p* = 10^−198^, 2-sided *t*-test) (Figure 2f). The spheroid viability profiles in OptiPASS^®^ medium stored for one day presented similar viability/mortality profiles to nonstored controls with mainly green cells at day 4 and day 14 (Figure 2g). In contrast, for the cultures stored for three days, spheroids presented a noncohesive and rare viable green cell population, for both days (day 4 and day 14) (Figure 2g). Finally, in OptiPASS^®^ medium, spheroid controls presented an increasing metabolic activity over time, with 16.1 ± 1.6 × 10^3^ UF at day 3, 22.4 ± 3.4 × 10^3^ UF at day 7 and 38.9 ± 4.5 × 10^3^ UF at day 14 (*p* = 10^−3^ and 10^−6^) (Figure 2h). Similarly, spheroids stored for one day presented an increasing metabolic activity during the culture time—16.1 ± 1.6 × 10^3^ UF, 21.3 ± 8.1 × 10^3^ UF and 22.5 ± 11.6 × 10^3^ UF at D3, D7 and D14, respectively (Figure 2h). In contrast, after three days of cold and normoxic storage, OptiPASS^®^ cultured spheroids presented a decreased metabolic activity at 7.8 ± 1.3 × 10^3^ UF at D14 compared to 16.1 ± 1.6 × 10^3^ UF, before the storage step (Figure 2h).

All these results suggest a clear loss of spheroid proliferation capacity, cell viability and cell metabolic activity, after one- or three-day cold and normoxic storage in RPMI 1640 medium. In contrast, in OptiPASS^®^ medium culture, these parameters seemed to be partially preserved for only one day of cold and normoxic storage of MDA-MB-231 spheroids.

### 3.2. Oxygen-Free and Cold Storage Combination Impact on MDA-MB-231 Spheroid Preservation in RPMI1640 and OptiPASS^®^ Culture Media

In order to achieve cold and oxygen-free conditions, spheroids in microplates were first cultured for 3 days under normal conditions in both RPMI1640 and OptiPASS^®^ media and were then hermetically packed using the Anaerogen™ oxygen-consumer. Spheroids were then stored for three days at 4 °C. Then, microplates were incubated in 37 °C and 5% CO_2_ and spheroid recovery was analyzed in the same conditions as previously presented.

**Figure 3:**
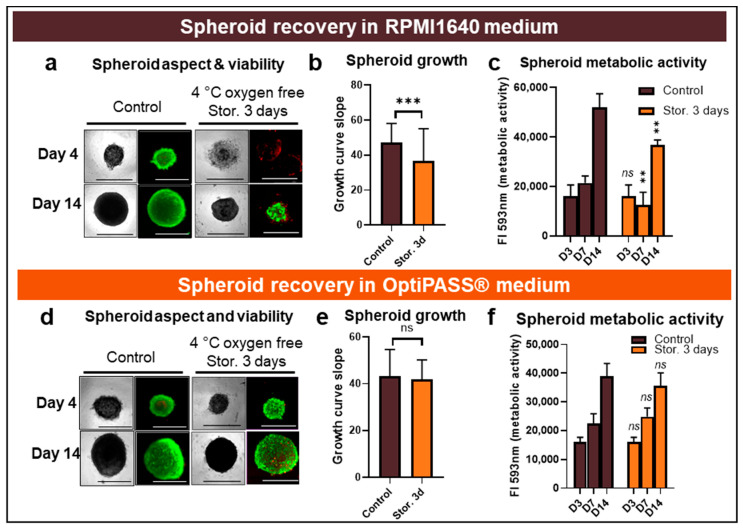
MDA-MB-231 spheroid preservation study after three-day storage at 4 °C and oxygen-free conditions in RPMI1640 and OptiPASS^®^ media culture. Three-day-old spheroids cultured in normal culture conditions, i.e., 37 °C, 5% CO_2_ in RPMI1640 or OptiPASS^®^ media culture, were exposed to oxygen-free conditions for 3 days at 4 °C. Then, the cultures were exposed again to normal culture conditions in which spheroid recovery was analyzed at day 3, day 4 and day 14. Spheroid integrity aspect and size (**a**) in RPMI and (**d**) in OptiPASS^®^ were analyzed with bright field microscopy and object size algorithm (Cytation™3MV, Gen5, BioTek^®^, M = 4X, scale bar = 1000 µm). Spheroid cell viability after storage was studied by Live/Dead^®^ tests (**a**) in RPMI and (**d**) in OptiPASS^®^ with green fluorescence, for viable cells and red fluorescence, as well as for dead cells, using Cytation™3MV equipped with GFP and IP fluorescence cubes (M = 4X, scale bar = 1000 µm). Spheroid cell proliferation was measured by the growth curve slope analysis between D4 and D14 (**b**) in RPMI and (**e**) in OptiPASS^®^. Spheroid metabolic activity after storage (**c**) in RPMI and (**f**) in OptiPASS^®^ medium culture was quantified using the resazurin tests. For each storage condition, significances compared to nonstored condition were indicated as ns (not significant; *p* > 0.05), ** *p* < 0.01, *** *p* < 0.001.

In RPMI 1640 medium, oxygen-free spheroids that were cold-stored for 3 days presented less cohesive structures than nonstored spheroids at D4 (Figure 3a). These spheroids evolved to a more compact aspect than controls at D14. However, the mean size of oxygen-free cold-stored spheroids (654.6 ± 212.2 µm) was significantly lower than nonstored controls at D14 at 913.2 ± 73.3 µm (*p* = 10^−10^, 2-sided *t*-test). Viability/mortality spheroid cell analysis by live/dead tests showed, for oxygen-free cold-stored spheroids, an absence of viable green cells at D4 and a low proportion of viable green cells at D14 (Figure 3a). In contrast, for nonstored spheroid controls compact green spheroids were detected at D4 and D14 (Figure 3a). In parallel, the growth curve slope for oxygen-free cold-stored spheroids in RPMI1640 was lower than nonstored controls at 36.6 ± 18.5 (compared to 47.3 ± 10.8, *p* = 10^−8^, two-sided *t*-test) (Figure 3b). Finally, metabolic activities of spheroids, assessed with resazurin tests after the oxygen-free cold storage, were 12.4 ± 5.2 × 10^3^ UF at D7 and of 36.8 ± 2.0 × 10^3^ UF at D14 (Figure 3c). These values remained lower than those obtained for nonstored controls equal to 21.4 ± 2.8 × 10^3^ UF at D7 and 52.0 ± 5.5 × 10^3^ UF at D14 (*p* = 10^−2^ and 10^−3^, 2-sided *t*-test).

In OptiPASS^®^ culture medium, after an oxygen-free 3-day long cold storage step, compact and cohesive spheroids, comparable to nonstored controls, were detected with bright field microscopy at D4 and D14 (Figure 3d). The mean sizes of stored OptiPASS^®^ cultured spheroids were 473.2 ± 50.2 µm at D4 and 881.5 ± 50.5 µm at D14. These results were in the same value range as nonstored controls sizes with sizes of 508.3 ± 101.1 µm at D4 and 913.2 ± 73.3 µm at D14 (*p* = 10^−7^ and 10^−3^, 2-sided *t*-test). Live/Dead^®^ tests showed a vast majority of green/viable cells giving similar viability profiles to nonstored controls (Figure 3d). Growth curve slopes between D4 and D14 were 41.7 ± 8.5 for oxygen-free cold-stored spheroids and comparable to nonstored controls at 43.1 ± 11.6 (*p* = 0.3, 2-sided *t*-test), showing similar proliferation rates between the two conditions (Figure 3e). Similarly, the metabolic activity evolution for oxygen-free cold storage, with 24.8 ± 3.0 × 10^3^ UF at D7 and 35.6 ± 4.5 × 10^3^ UF at D14, remained close to nonstored control spheroids, with 22.4 ± 3.4 × 10^3^ UF at D4 and 38.9 ± 4.5 × 10^3^ UF at D14 (*p* = 0.3 and 0.3, 2-sided *t*-test).

All these results showed that in OptiPASS^®^ medium, the combination of oxygen-free and cold storage over 3 days made it possible to preserve the properties of the MDA-MB-231 spheroids in comparison to nonstored controls. This last condition for the storage of MDA-MB-231 spheroids had no impact on these culture parameters, and longer storages in OptiPASS^®^ medium were then studied.

### 3.3. OptiPASS^®^ Synthetic Medium Allows Longer Cold and Oxygen-Free Storage of MDA-MB-231 Spheroids without Changing Model’s Phenotype

OptiPASS^®^ medium cultured MDA-MB-231 spheroids were maintained for 3 days under normal conditions and then exposed to oxygen-free conditions at 4 °C for 5, 7, 14 or 30 days. After each storage, microplates were exposed to 37 °C and 5% CO_2_. This is the same as previous parameters—i.e., spheroid integrity, growth, viability/mortality profile, metabolic activity and also ki67 proliferation marker expression—and spheroid hypoxia levels were analyzed at different times in comparison to nonstored spheroid controls.

**Figure 4:**
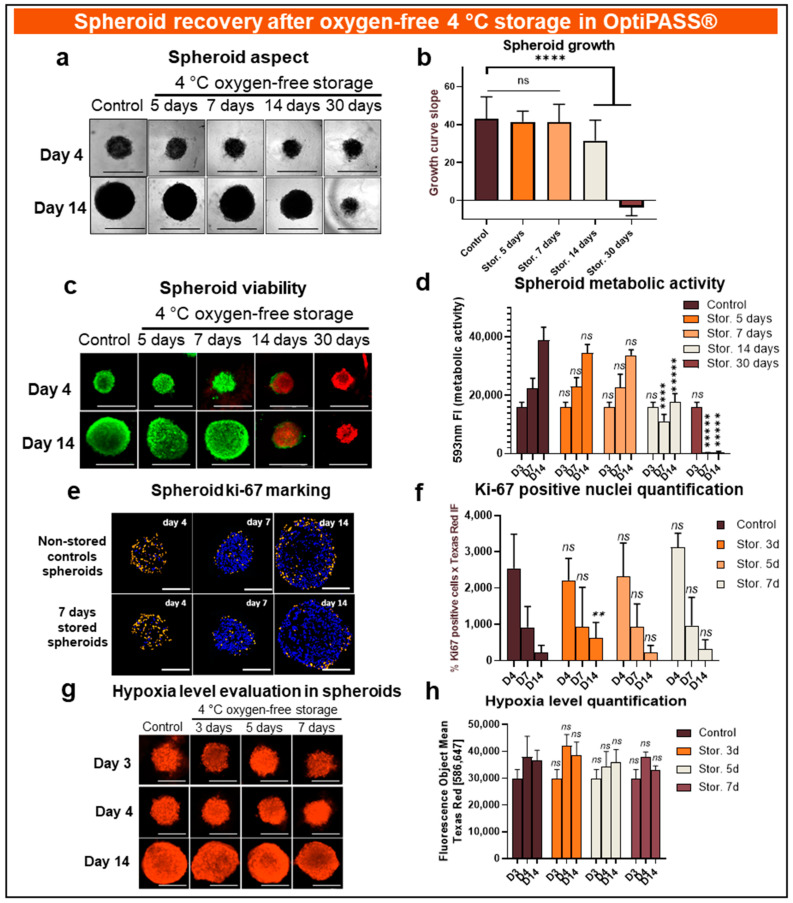
MDA-MB-231 spheroid preservation analysis after several days of storage in oxygen-free and 4 °C storage in OptiPASS^®^ medium culture. Three-day-old spheroids cultured in normal culture conditions, i.e., 37 °C, 5% CO_2_ in OptiPASS^®^ medium culture were exposed to oxygen-free conditions at 4 °C for 5, 7, 14 or 30 days. After each storage time, spheroid recovery was analyzed in comparison to nonstored control spheroids. Samples’ integrity aspects and sizes (**a**) were studied with bright field microscopy and object size algorithm (Cytation™3MV, Gen5, BioTek^®^, M = 4X, scale bar = 1000 µm). Growth curve slopes were calculated using spheroids’ size values between the 4th and the 14th days of culture, reflecting recovery of proliferation capacities after storage step (**b**). Spheroid viability after storage was assessed by Live/Dead^®^ tests showing viable cells (green fluorescence) and dead cells (red fluorescence) using Cytation™3MV equipped with GFP and IP fluorescence cubes (M = 4X, scale bar = 1000 µm) (**c**). Complementary to this, spheroid metabolic activity was quantified using resazurin tests (**d**). Otherwise, spheroid tumoral proliferation gradient analysis was carried out by ki67 immunostaining at D4, D7 and D14 and imaged with Texas Red filter (M = 10X, scale bar = 200 µm) on Cytation™3MV instrument (BioTek^®^). Ki67-positive nuclei (in orange) were identified among other nuclei (in blue) with adapted algorithm on Gen5 software (BioTek^®^, pictures showed for nonstored controls and spheroids stored for 7 days) (**e**). Global ki67-expression was quantified and compared between nonstored controls and 3-, 5- and 7-day-long cold and oxygen-free storage conditions in OptiPASS^®^ medium (**f**). Finally, spheroid hypoxia level was studied using ROS-ID^®^ kit staining at D3, D4 and D14 (**g**,**h**). Spheroids were imaged with Cytation™3MV equipped with Texas Red fluorescence cube (M = 4X, scale bar = 500 µm) (**g**). Hypoxia level was quantified by acquired fluorescent signal intensity with Gen5 software (BioTek^®^) (h). For each storage condition, significances compared to nonstored condition were indicated as ns (not significant; *p* > 0.05), ** *p* < 0.01, **** *p* < 0.0001, ***** *p* < 0.00001.

First, concerning spheroid topology in terms of cell mass cohesion and cellular compaction, no difference was detected at D4 between the spheroids stored for 5 and 7 days and the nonstored controls with compact and ovoid spheroids (Figure 4a). In contrast, the spheroids stored for 14 and 30 days presented less cohesive shapes than the control nonstored spheroids with less regular borders (Figure 4a). Mean sizes of spheroids at D4 (just after return in culture conditions) were 466.3 ± 3.5 µm, 449.7 ± 29.9 µm, 367.2 ± 35.8 µm and of 465.1 ± 64.9 µm for spheroids stored for 5, 7, 14 and 30 days, respectively, compared to 508 ± 101.1 µm for nonstored controls (*p* = 10^−9^, 10^−18^, 10^−44^ and 10^−7^, 2-sided *t*-test). Otherwise, at D14, the spheroids stored for 5, 7 and 14 days showed regularly round and cohesive shapes with a mean size in the same range as nonstored controls—831.3 ± 51.3 µm, 845.0 ± 70.8 µm and 825.5 ± 140.2 µm, respectively, compared to 908.1 ± 52.1 µm for controls (*p* = 10^−24^, 10^−13^ and 10^−8^, 2-sided *t*-test). For 30-day storage, spheroids presented a compaction loss aspect (Figure 4a) with a mean size greatly inferior to control values at day 14 (421.0 ± 54.9, *p* = 10^−227^, 2-sided *t*-test).

Then, to characterize spheroids’ proliferation poststorage recovery, the growth curve slopes were analyzed from D4 to D14. For spheroids stored for 5 and 7 days, slopes were at 41.5 ± 5.5 and 41.3 ± 9.5, respectively, similar to the nonstored control value of 43.1 ± 11.6 (*p* = 0.09 and 0.07, 2-sided *t*-test) (Figure 4b). However, significant differences were detected between control values and slopes of spheroids stored for 14 days (31.5 ± 10.9, *p* = 10^−12^, 2-sided *t*-test) and of spheroids stored for 30 days (-3.7 ± 4.3, *p* = 10^−208^, 2-sided *t*-test) (Figure 4b). In parallel, Live/Dead^®^ tests carried out at D4 and D14 revealed a majority of green fluorescent viable cells comparable to nonstored controls after 5 and 7 days of cold storage (Figure 4c). For experiments with longer storage times (14 and 30 days), red dead cells combined with a persistent green cell population were detected for spheroids stored for 14 days and exclusively read dead cells for samples stored for 30 days (Figure 4c).

These viability profiles were then confirmed with the resazurin metabolic activity test performed on D3, D7 and D14. The spheroids stored for 5 and 7 days presented metabolic activity evolutions similar to nonstored controls (Figure 4d), with fluorescent intensities of 23.0 ± 3.0 × 10^3^ at D7 and 34.4 ± 3.0 × 10^3^ at D14 for spheroids stored for 5 days compared to 22.4 ± 3.4 × 10^3^ and 38.9 ± 4.5 × 10^3^ for nonstored spheroid controls (*p* = 0.82 and 0.13, 2-sided *t*-test). However, the spheroids stored for 14 days showed significantly decreased metabolic activity, with 11.1 ± 2.4 × 10^3^ at D7 and 17.6 ± 3.0 × 10^3^ at D14 (*p* = 10^−^^4^ and 10^−^^5^, 2-sided *t*-test). Finally, the results obtained for the spheroids stored for 30 days were dramatically lower than those of nonstored control and close to blank background (0.3 ± 0.5 × 10^3^ and 0.4 ± 0.3 × 10^3^ for D7 and D14, respectively, *p* = 10^−^^4^ and 10^−^^5^, 2-sided *t*-test), presenting an almost total loss of spheroid viability after the storage step.

Regarding the absence of an impact of 3-, 5- and 7-day-long cold and oxygen-free storage on OptiPASS^®^ cultured MDA-MB-231 spheroids in terms of proliferation, viability and metabolic activity, phenotypic characterization was then studied for these three storage conditions in comparison to nonstored control conditions.

Firstly, ki67 expression was analyzed by immunostaining on spheroid cuts. For all tested conditions, specific staining was achieved showing the presence of orange intense ki67-positive nuclei (Figure 4e). Ki67-positive nuclei were distributed in all spheroid areas at D3 and D7, while they were rather located in spheroid borders at D14 (Figure 4e). The spheroid ki67 global expression (% ki67-positive cells x fluorescence intensity) was then analyzed (Figure 4f). For nonstored controls, ki67 expression decreased from 25.4 ± 9.4 × 10^2^ UF at D4, to 9.2 ± 5.7 × 10^2^ UF at D7 and to 0.2 ± 0.2 × 10^2^ UF at D14. This evolution was similar to those seen for spheroids stored for 3 days, with values of 22.2 ± 6.0 × 10^2^ UF at D4 (*p* = 0.4, 2-sided *t*-test), 9.4 ± 1.1 × 10^2^ UF at D7 (*p* = 0.9, 2-sided *t*-test) and 6.2 ± 4.3 × 10^2^ UF at D14 (*p* = 0.01, 2-sided *t*-test). These results were also similar for spheroids stored for 5 and 7 days spheroids (Figure 4f). Secondly, the spheroid hypoxia level was evaluated using a ROS-ID^®^ fluorescence kit. For all tested conditions, fluorescent staining was homogeneously distributed on all spheroid surfaces (Figure 4g). The spheroid hypoxia level remained relatively stable over time for nonstored controls with a mean of 30.0 ± 3.3 × 10^3^ UF at D3, of 37.8 ± 7.8 × 10^3^ UF at D4 and of 36.6 ± 3.8 × 10^3^ UF at D14 (Figure 4h). Similar values were obtained after spheroid storage with 42.1 ± 4.2 × 10^3^ UF, 34.5 ± 5.5 × 10^3^ UF and 37.8 ± 2.0 × 10^3^ UF at D4 for 3-, 5- and 7-day-long storage conditions, respectively (*p* = 0.20, 0.40 and 0.99, 2-sided *t*-test) (Figure 4h). In the same way, at D14, the stored spheroids’ hypoxia levels were similar to those obtained for nonstored control with 38.5 ± 5.0 × 10^3^ UF, 35.9 ± 4.8 × 10^3^ UF and 33.1 ± 1.5 × 10^3^ UF for 3-, 5- and 7-day-long storage conditions, respectively (*p* = 0.80, 0.98 and 0.35, 2-sided *t*-test) (Figure 4h).

These results suggest that OptiPASS^®^ synthetic medium in oxygen-free and cold conditions had the ability to preserve MDA-MB-231 spheroids for a maximum time of 7 days. For the studied parameters, under these storage conditions, spheroids would have the same characteristics as nonstored control spheroids in terms of proliferation, viability metabolic activity, ki67 expression rate and hypoxia level.

### 3.4. Anticancer Drug Sensitivity Evaluation of MDA-MB-231 Spheroids After Cold and Oxygen-Free Storage in OptiPASS^®^ Medium Culture

In order to evaluate the drug sensitivity of MDA-MB-231 spheroids after oxygen-free 4 °C storage for 3, 5 or 7 days in OptiPASS^®^ medium, these spheroids were treated with two conventional chemotherapeutics—i.e., epirubicin and cisplatin at 0.1, 1 and 10 µM or two anti-PARP targeted chemotherapeutics—i.e., olaparib and veliparib at 0.5, 5 and 50 µM. Drug cytotoxicity action on spheroids was assessed by the analysis of growth curve slopes, spheroid viability/mortality profile and cell metabolic activity in comparison with nonstored control spheroids.

**Figure 5:**
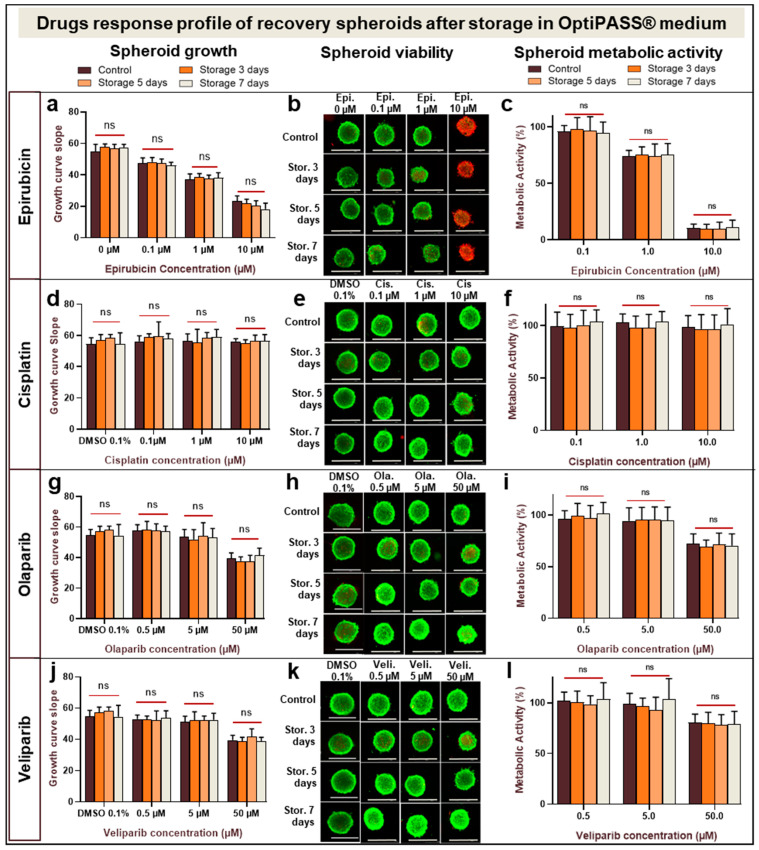
Anticancer drug sensitivity evaluation on recovered MDA-MB-231 spheroids after 4 °C and oxygen-free storage in OptiPASS^®^ medium. Three-day-old spheroids cultured in normal culture conditions, i.e., 37 °C, 5% CO_2_ in OptiPASS^®^ medium culture, were stored at 4 °C in oxygen-free conditions for 3, 5 or 7 days. After returning to normal culture conditions for 2 days, spheroids were treated with epirubicin (0.1, 1 or 10 µM), cisplatin (0.1, 1 or 10 µM), olaparib (0.5, 5 or 50 µM) or veliparib (0.5, 5 or 50 µM) up to D10. For each condition, growth curve slopes during treatment were calculated for epirubicin (**a**), cisplatin (**d**), olaparib (**g**) and veliparib (**j**). Live/Dead^®^ test profiles were imaged with plate reader Cytation™3 MV (GFP and IP filters-M = 4X) for epirubicin (**b**), cisplatin (**e**), olaparib (**h**) and veliparib (**k**). Metabolic activity rates were evaluated with resazurin test (Cytation™3MV-fluorimetry 593nm) for epirubicin (**c**), cisplatin (**f**), olaparib (**i**) and veliparib (**l**). ns = *p* > 0.05.

When the epirubicin concentration increased, dose-dependent decreases in growth curve slopes was detected for nonstored spheroids with 54.8 ± 4.7 for control, 47.5 ± 3.3 for 0.1 µM, 36.9 ± 3.7 for 1 µM and 23.3 ± 3.3 for 10 µM of epirubicin, respectively (Figure 5a). The same decreases in growth of curve slopes were observed for spheroids cold-stored for 3, 5 and 7 days with pooled data of 57.3 ± 2.2, of 47.1 ± 2.7, of 37.9 ± 2.7 and of 20.1 ± 3.6 for control, 0.1, 1 and 10 µM epirubicin concentrations, respectively (*p* ≥ 0.9, >0.9, 0.9 and >0.9, ANOVA) (Figure 5a). In parallel, for the three storage conditions similar spheroid viability/mortality profiles were observed with a majority of viable green cells after 0.1 and 1 µM epirubicin treatments and a large proportion of red dead cells after 10 µM epirubicin treatment (Figure 5b). The same profiles were observed for nonstored controls. For the metabolic activity parameter, a dose-dependent decrease was also detected in presence of increasing epirubicin concentration. Indeed, the nonstored epirubicin treated spheroids presented metabolic activities of 95.8 ± 5.4% with 0.1 µm, of 73.9 ± 5.5% with 1 µM and of 10.4 ± 3.6% with 10 µM of this drug (Figure 5c). Pooled data of spheroids stored for 3, 5 or 7 days also presented the same metabolic activity evolutions as nonstored spheroids, with 96.6 ± 10.5%, 75.0 ± 9.0% and 10.2 ± 5.5% for 0.1, 1 and 10 µM epirubicin, respectively (*p* ≥ 0.9, ANOVA). Similarly, the epirubicin IC_50_ values were 2.19 µM for nonstored spheroid controls and 2.29, 2.20 and 2.29 µM for 3-, 5- and 7-day-long storage conditions, respectively.

After 0.1, 1 and 10 µM cisplatin treatments of nonstored spheroids, the curve slopes remained unchanged at 56.2 ± 3.6, 56.4 ± 4.6, and 56.3 ± 1.7, respectively, similar to the 0.1% DMSO control spheroids at 54.6 ± 4.0 (*p* = 0.68, 0.74 and 0.56, ANOVA (Figure 5d)). Comparable curve slopes were also obtained for spheroids stored for 3, 5 and 7 days, with pooled data of 56.6 ± 5.1, 58.8 ± 5.6, 57.7 ±6.0 and 56.0 ± 3.5 for 0.1% DMSO control and 0.1, 1 and 10 µM cisplatin, respectively (*p* ≥ 0.9, ANOVA) (Figure 5d). Likewise, for nonstored spheroids and all stored spheroid conditions, the same Live/Dead^®^ test profiles with compact and viable cell masses were detected (Figure 5e). For metabolic activity analysis, no difference was detected between cisplatin treated nonstored spheroid controls and cisplatin treated spheroids stored for 3, 5 and 7 days. Pooled data were 100.8 ± 12.6%, 100.0 ± 11.3% and 98.2 ± 14.0% for 0.1, 1 and 10 µM cisplatin, respectively (*p* ≥ 0.9, ANOVA) (Figure 5f).

In presence of increasing olaparib concentrations, the growth curve slopes of MDA-MB-231 nonstored spheroids were 57.9 ± 3.7 for 0.5 µM and of 53.5 ± 5.0 for 5 µM, similar to 0.1% DMSO treated nonstored control spheroids at 54.6 ± 4.0 (*p* = 0.36 and 0.93, ANOVA). In contrast, with 50 µM anti-PARP1 growth curve slopes decreased significantly to 39.4 ± 3.7 (*p* = 10^−5^, ANOVA) compared to DMSO control (Figure 5g). The same results were observed with spheroids stored for 3, 5 and 7 days with pooled data of 56.6 ± 5.1, 57.7 ± 4.5, 53.1 ± 7.0 and 39.0 ± 4.4 after treatment with 0.1% DMSO control and 0.5, 5 and 50 µM olaparib, respectively (*p* ≥ 0.9, ANOVA) (Figure 5g). Live/Dead^®^ fluorescent analysis presented the same green compact cell masses in stored and nonstored as well as treated or nontreated conditions (Figure 5h). After olaparib treatment of nonstored spheroids, metabolic activity rates were similar for 0.5 and 5 µM, at 96.0 ± 8.4% and 94.4 ± 12.8% (*p* = 0.91, ANOVA). Otherwise, for 50 µM olaparib metabolic activity decreased significantly to 72.3 ± 9.7% (*p* = 10^−3^, ANOVA) (Figure 5i). Comparable results were obtained for spheroids stored for 3, 5 and 7 days with pooled data of 99.5 ± 11.6%, 95.0 ± 12.3% and 70.3 ± 9.8% after 0.5, 5 and 50 µM anti-PARP1 treatments, respectively (*p* ≥ 0.9, ANOVA) (Figure 5i). These data allowed us to estimate the Olaparib IC_50_ doses, which were 127.9, 111.3, 123.8 and 117.9 µM for nonstored controls and spheroids stored for 3, 5 and 7 days, respectively.

Finally, after veliparib treatment, nonstored spheroid growth curve slopes were 54.6 ± 4.0 for 0.1% DMSO control and similar to 52.9 ± 2.6 with 0.5 µM (*p* = 0.46, ANOVA) and to 51.4 ± 3.5 with 5 µM (*p* = 0.21, ANOVA). For 50 µm of this anti-PARP1, growth curve slopes decreased significantly to 39.4 ± 3.1 (*p* = 10^−5^, ANOVA) (Figure 5j). Then, Live/Dead^®^ tests of veliparib treated spheroids presented viable green cell masses for all concentrations and all storage conditions (Figure 5k). The nonstored spheroid metabolic activity rates were 101.7 ± 8.8% for 0.5 µM, of 99.0 ± 10.4% for 5 µM and of 80.7 ± 8.2% for 50 µM. They were comparable to the values obtained for 3-, 5- and 7-day-long storage conditions with pooled data of 101.1 ± 12.0%, 97.8 ± 14.8% and 78.9 ± 11.0% after 0.5, 5 and 50 µM veliparib treatments, respectively (*p* = >0.9, ANOVA) (Figure 5l). Close values of estimated veliparib IC_50_ doses were obtained with 212.0, 197.6, 173.6 and 195.8 µM for nonstored controls and spheroids stored for 3, 5 and 7 days, respectively.

These results indicated different MDA-MB-231 spheroid drug responses according to the selected anticancer agent. Indeed, a strong cytotoxic effect was detected after epirubicin treatment, while no effect was detected after cisplatin treatment. For both anti-PARP1 targeted therapy drugs, only the highest-tested concentration had an impact on spheroid growth and metabolism activity. Otherwise, and very interestingly, spheroid drug responses were similar between nonstored spheroids and 3-, 5- or 7-day-long oxygen-free cold-stored spheroids cultured in OptiPASS^®^. This suggests that selected spheroids storage conditions in OptiPASS^®^ medium do not impact MDA-MB-231 spheroids’ anticancer drug sensitivity.

## 4. Discussion

Spheroid in vitro models remain particularly relevant for preclinical evaluation of anticancer treatments, combining production reproducibility and predictivity of response at the same time [28]. Their use is more and more solicited in the drug development process, especially for aggressive tumoral types such as TNBCs [29]. They allow rapid investigation of drug response in terms of heterogeneity of metabolic cell layers and drug penetration inside cell masses [14]. In addition, these models are of great interest as they allow the use of animals to be reduced, refined and replaced (rule of 3Rs) during the long, expensive and exhausting drug development process [17,30]. Moreover, recent advances in 3D culture formation have enabled scientists to replicate models of avascular tumor in high number with acceptable reproducibility applicable for scalable and large range preclinical trials. Another key factor for in vitro modeling using 3D models is the culture medium [19]. In this way, our previous works focused on the development of two TNBC spheroid models using MDA-MB-231 and SUM1315 cell lines in the new serum-free culture medium entitled OptiPASS^®^ [23]. Our results highlighted a clear repeatability and reproducibility of spheroid formation in these experimental conditions. Furthermore, cell proliferation, viability, and drug sensibility, for both MDA-MB-231 and SUM1315 models, were clearly optimized in OptiPASS^®^ medium. These results also highlight the key role of the culture medium in the optimization of the culture model’s predictive response [23]. This approach is relevant for improving drug sensibility threshold in spheroids, thereby making drug screening more efficient.

To optimize the use of a high number of 3D culture models in high throughput screening assays, a concept of spheroid storage should be considered. In this field, cryopreservation methods represented the gold standard to maintain the viability/integrity of biological samples for several years or decades. However, in the case of 3D culture models, these techniques are time consuming, difficult to apply and often require specific equipment. Moreover, the compulsory use of cytotoxic cryoprotectants causes a partial cell viability recovery after thawing [31,32]. Thereby, in the last few years, other alternative methodologies such as hypothermia, hypoxia and synthetic serum-free medium conditions, leading to biological properties preservation of various cell types, have been developed. Indeed, hypothermic storage, i.e., cold temperature close to 4°C, was successfully used to preserve pluripotent stem cell-derived cardiomyocytes, bone marrow-derived mesenchymal stem cells or hepatocytes. In fact, hypothermia allows the majority of biological processes to be stopped and thus force cells to enter dormant states. This phenomenon is crucial to limit cell damages and arrest their evolution during the storage time [33,34,35]. Hypoxia was also described to prevent oxidative stress and the formation of ROS in the culture medium at 4 °C to preserve red blood cells [36]. Likewise, synthetic serum-free media have been shown to play a role in preservation of multipotent mesenchymal stromal cells at 4°C [37]. More recent works carried out on 3D cell culture models also showed that the combination of cold (4 °C) and anoxic storage conditions, but in presence of serum-supplemented culture medium, allowed the preservation of colorectal multicellular tumor spheroids for 18 days [24]. These works revealed the importance of the decrease in oxygen pressure to reversibly arrest spheroid growth while maintaining their principal characteristics [38]. 

In this context, with the objective of developing a new storage concept of 3D cell culture models, our works were focused on the development of a spheroid storage methodology using the TNBC MDA-MB-231 cell line as a model in the new synthetic OptiPASS^®^ medium, in comparison with RPMI FBS-supplemented medium preconized for this cell line.

For this, several culture conditions were studied. Indeed, the spheroids were cultured in both tested medium cultures in microplates under conventional conditions at 37 °C in each culture medium for 3 days. Then, they were stored at 4 °C with or without anoxia for 1, 3, 5, 7, 14 or 30 days. After each conservation period, spheroids were placed back under 5% CO_2_ conditions at 37 °C. Spheroid recovery was evaluated with several parameters such as (i) spheroid growth via spheroid growth curve slopes between day 4 and day 14, (ii) cell viability via live/dead test and (iii) metabolic activity via resazurin test.

Firstly, the 3-day storage at 4 °C in normoxic conditions in both RPMI1640 and OptiPASS^®^ culture medium led to an incomplete preservation of spheroid growth curve slopes (decreases of 93.7 and 101.5%) and metabolic activity (restauration values of 1.9 and 20.1%), respectively. Indeed, the cold-only storage condition is far from sufficient to obtain usable spheroids for further experiments.

Secondly, in order to optimize the spheroid storage step in both media, spheroids were placed in an oxygen-free environment at 4 °C for 3 days, according to previous works of Gomes et al. [24]. The results showed that this condition led to an improvement of spheroid integrity in RPMI medium for 14 days. However, the cell proliferation and cell metabolic activity were only partially restored (recoveries of 77.4 and 70.8%, respectively). In contrast, 3 days of cold (4 °C) and oxygen-free storage in OptiPASS^®^ medium preserved the properties of the MDA-MB-231 spheroids. Indeed, in this condition, between day 4 and day 14, no impacts on spheroid integrity in growth curve slopes (recovery of 96.8%) and cell metabolic activity (preservation of 91.5%) were detected. All these results show that the properties of the MDA-MB-231 spheroids can be preserved after their storage for 3 days in OptiPASS^®^ medium at 4 ° C and under oxygen-free conditions for further recovery.

Then, in order to optimize the conservation in OptiPASS^®^ medium at 4 °C and in oxygen-free conditions, longer storage times of 5, 7, 14 and 30 days were analyzed in this medium. Interestingly, after 5- and 7-day-long cold and oxygen-free storage in OptiPASS^®^, the spheroid growth curve slopes (recoveries of 96.2 and 95.6%, respectively) and the metabolic activity (recoveries of 91.3 and 88.4%, respectively) were preserved compared to nonstored controls, confirming an absence of impact on this spheroid growth parameter. In contrast, the longer storage times, i.e., 14 and 30 days, did not allow spheroid growth curve slopes (recoveries of 73.1 and −0.1%, respectively) and metabolic activity (decreases of 45.2 and 99.9%, respectively) to be maintained compared to nonstored controls. Therefore, in MDA-MB-231 spheroid models, cell proliferation, cell viability and cell metabolic activity parameters may be preserved after storage times of 3, 5 or 7 days in OptiPASS^®^ medium at 4 °C and in oxygen-free conditions. This conservation methodology was then selected for the following experiments.

MDA-MB-231 spheroid recovery performances were then assessed with ki67 expression rate and spheroid global hypoxia level analyses, reflecting the cell proliferation capacity and global hypoxia status [30,31]. After 3, 5, and 7 days of storage, the ki67 expression rate recoveries on spheroids at day 4 were 87.2, 90.9 and 122.5%, respectively, in comparison to nonstored controls. Similarly, spheroid hypoxia level was not disturbed by the storage step with hypoxia rate recovery at day 14 of 111.4, 91.2 and 99.8%, respectively. These results suggest that-tested storage conditions had no influence on these parameters and thus on tumoral proliferation gradients and hypoxia levels of MDA-MB-231 spheroid models. Indeed, the maintenance of these parameters was significant as they may influence the spheroid response in drug screening.

Thereafter, to ensure that the storage step did not affect spheroids sensibility threshold to anticancer drugs, the spheroid drug response was analyzed. For this, MDA-MB-231 spheroids, after 3, 5 and 7 days of storage in OptiPASS^®^ medium at 4 °C and in oxygen-free conditions, were treated with increasing concentrations of two classic chemotherapies, i.e., epirubicin or cisplatin, and two PARP1 inhibitors—i.e., olaparib or veliparib. In presence of various drugs, whatever the storage conditions, recovered spheroids presented similar cell proliferation decreases (57.5 and 64.1% with 10 µM epirubicin, 0.2 and 1.1% with 10 µM cisplatin, 32.0 and 32.6% with 50 µM olaparib and 23.7 and 27.8% with 50 µM veliparib) and cell metabolic activity decreases (96.2 and 89.4% with 10 µM epirubicin, 2.6% and 0.3% with 10 µM cisplatin, 29.7 and 27.7% with 50 µM olaparib and 20.6 and 22.4% with 50 µM veliparib) compared to nonstored controls. All these results indicate that this new methodology combining a spheroid storage at 4 °C and oxygen-free atmosphere in OptiPASS^®^ medium does not influence spheroid responses to various drugs in terms of growth, viability, and metabolic activity. In terms of the unchanged model’s behavior and drug sensitivity profiles, stored spheroids can be used in drug screening tests the same way as nonstored spheroids.

In our experimental conditions, the comparison of both media culture performances highlighted that our innovative spheroid storage concept is principally permitted thanks to the new OptiPASS^®^ serum-free medium, in comparison to serum-supplemented media. This culture medium with an original confidential formulation (developed by BIOPASS company) probably plays a major role in the spheroid recovery process. Indeed, serum-free media enriched in antioxidants substrates have already been described as improving the preservation of stromal stem cell monolayer cultures at 4 °C [37]. Otherwise, other parameters specific to cell type models such as energy metabolism may also play a role to conserve biological characteristics. Indeed, energy metabolism reprogramming is a significant hallmark of cancer cells [39]. For basal-like cancer cell line models, such as MDA-MB-231, mitochondrial defects are described compared to other breast cancer subtypes [40]. This glycolytic switch t the origin of the differences in metabolism between tumoral and nontumoral cell lines. However, cold and oxygen-free conditions have been already used successfully for the conservation of different cell types such as cardiomyocytes and mesenchymal stem cells presenting upregulated mitochondrial respiration [33,34,37].

Furthermore, this new methodology of storage spheroids was relatively simple to apply and did not require expensive specific equipment. Especially, there is no need to transfer spheroid models to different supports or to change the cell culture medium. This point may be very important, especially for high added-value models, allowing the reduction in spheroid manipulations and thus limiting spheroid disturbances or losses. Moreover, the oxygen-free atmosphere generating device allows the induction of anoxic conditions very quickly and reproducibly. Indeed, once the system is closed, oxygen pressure decreases to 0% after less than 20 min (Appendix A). This concept could be easily applied to spheroids from others cancer cell lines. Moreover, another great interest of this methodology is that during optimized storage conditions, spheroid size did not evolve during storage time in OptiPASS^®^ medium. Indeed, the spheroid size ratio measured between the day when spheroids were placed at 4 °C and the day after their return in culture incubator was close to 1 (1.02 ± 0.09, 0.94 ± 0.08 and 1.05 ± 0.15 for 3, 5 and 7 days of storage, respectively), while it was of 1.22 ± 0.18 for nonstored controls (*p* < 0.0001). Thereby, this methodology allowed the cessation of spheroid growth while maintaining their ability to restart after their return to normal cell culture conditions. It should be considered as a break in spheroid culture time, allowing the MDA-MB-231 model to be preserved for up to 7 days. This mechanism can be related to the tumoral cellular quiescence phenomenon. Indeed, the stress caused by cold and oxygen-free conditions may have induced spheroid cell entry in dormancy, which is at the origin of a reversible arrest of spheroid growth. Moreover, cellular quiescence generally occurs in response to stress or to a hostile environment and drives cancer cells to enter in a dormant state, corresponding to a reversible cell cycle arrest in order to survive and being reactivated in favorable conditions [41]. This mechanism is known in cancer biology to initiate metastases and generate resistance mechanism to anticancer therapies [42,43]. This approach could turn out to be very relevant for modelling cancer dormancy. Indeed, it can represent a way to artificially recreate avascular tumor-like dormant cell masses useful for testing dormancy-targeting drugs in development. For example, several epigenetic-targeting drugs, including inhibitors of histone lysine demethylase, have been developed to eradicate tumoral dormant cells [44]. This storage concept may be the starting point for the development of future cancer dormancy study models.

Otherwise, in our previous studies, the serum-free OptiPASS^®^ medium had already demonstrated the improvement in growth of TNBC spheroids while maintaining their drug sensitivity in normal culture conditions [23]. Moreover, these specific works highlight the capacity of this medium to (i) preserve and (ii) relevantly recover spheroid models similarly to nonstored spheroids. These properties represent an innovative added value considering the use of this medium for preclinical models. Thus, this alternative concept to cryoconservation could be adapted to other tumoral or nontumoral multicellular models and also various clinical samples.

Therefore, this concept opens the way (i) to the development of new tumor dormancy models, (ii) to the preservation of precious biological materials in the laboratory, and also (iii) to easy handling during interlaboratory transport for several days. These works are currently underway.

## Figures and Tables

**Figure A1:**
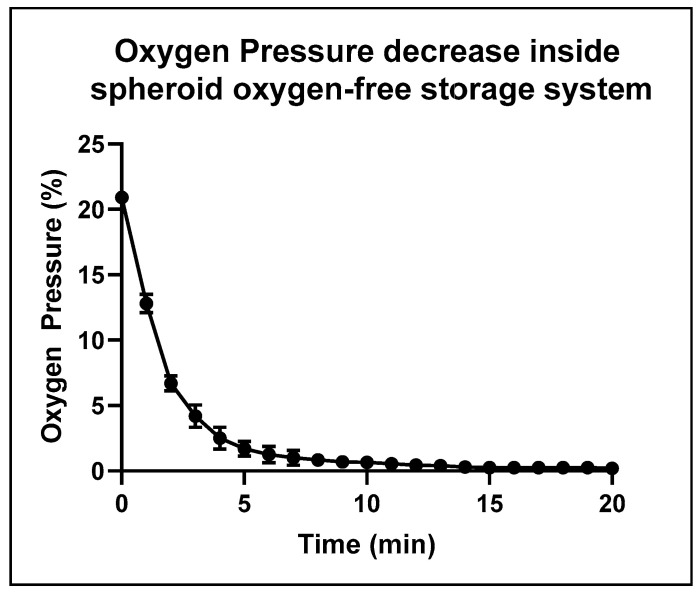
Validation of the oxygen-free atmosphere generating system.

## Data Availability

The data presented in this study are not publicly available and are available on request from the corresponding author.

## Supplementary Materials

The following are available online at https://www.mdpi.com/article/10.3390/cancers13081945/s1, Figure S1: Validation of the staining with the ROS-ID kit orange reagent (EnzoLifeSciences catalogue #ENZ-51042) to evaluate MDA-MB-231 spheroid hypoxia level.

## Appendix A

In order to validate oxygen-free atmosphere generating system for spheroid storage, a microplate containing culture medium was packed in hermetically closed plastic bag (Flo™, Marcilly, France, catalogue #763783) with anaerobic gas generating AnaeroGen™ 2.5L (Oxoid™, Dardilly, France, catalogue #AG0025A) at 4 °C. Oxygen pressure was measured with a monogaz O2 detector (Honeywell, Charlotte, NC, USA) every minute for 20 min. Once the system was closed, the oxygen pressure rapidly decreased in the system, falling from 20.9 ± 0.0% at T0 (0 min) to 0.7 ± 0.4% after 10 min and to 0.1% ± 0.0% after 20 min. These values validate this system for artificially creating oxygen-free conditions for the storage of MDA-MB-231 spheroids.
