# Supplementary material for: The New Serum-Free OptiPASS® Medium in Cold and Oxygen-Free Conditions: An Innovative Conservation Method for the Preservation of MDA-MB-231 Triple Negative Breast Cancer Spheroids
Source: Cancers (Basel). 2021 Apr 18;13(8):1945. doi: 10.3390/cancers13081945 (PMC8073891; doi:10.3390/cancers13081945)
Supplement: Supplementary file 1 [file cancers-13-01945-s001.zip › cancers-1178939-supplementary.pdf]

Supplementary materials

The New Serum-Free OptiPASS® Medium in Cold and Oxygen-Free Conditions: An Innovative Conservation Method for the Preservation of MDA-MB-231 Triple Negative Breast Cancer Spheroids

Antoine Goisnard [1], Clémence Dubois [1,2], Pierre Daumar [1,+], Corinne Aubel [3,+], Marie Depresle [2], Jean Gauthier [2,4], Bernard Vidalinc [2,4], Frédérique Penault-Llorca [5], Emmanuelle Mounetou [1] and Mahchid Bamdad [1,*]

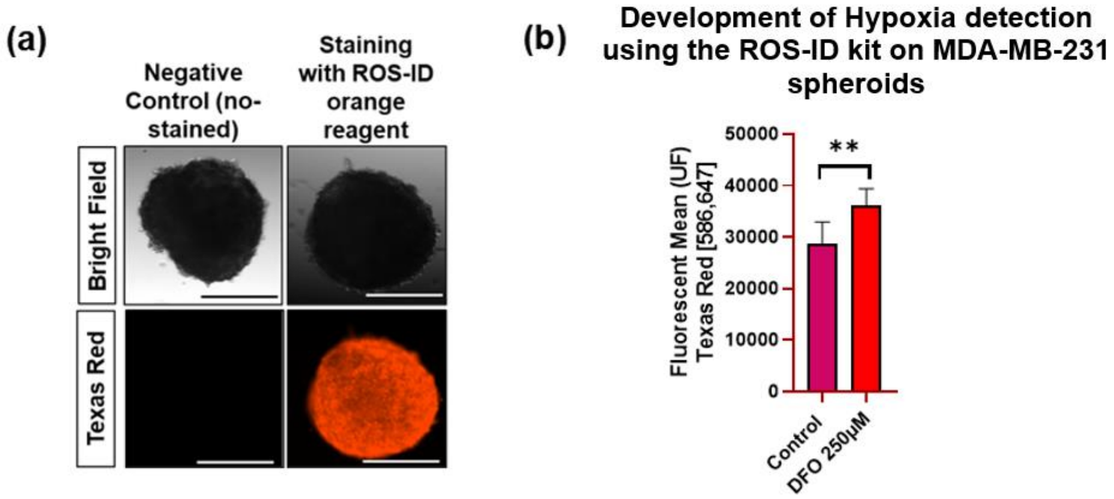

Figure S1: Validation of the staining with the ROS-ID kit orange reagent (EnzoLifeSciences catalogue #ENZ-51042) to evaluate MDA-MB-231 spheroid hypoxia level. To realise a negative control of the staining **(a)**, MDA-MB-231 spheroids cultured during 14 days in OptiPASS® medium were maintained unstained (negative control condition) or exposed with Hypoxia Red reagent at a concentration of 250 µM diluted in OptiPASS® medium for 4 hours (staining condition). Spheroids were transferred in a µ-Slide 8 Well (Ibidi, catalogue #µ-Slide 8 Well) to be imaged using the fluorescent microscopy module of the Cytation™3MV cell analyser (Biotek® - M=4X - fluorescence filter= Texas Red - scale bare=500 µm). For the negative control without staining, no specific fluorescent signal has been detected. In contrast, for condition with exposure to ROS-ID orange reagent, an orange fluorescent staining was detectable on all spheroid area. In complement, a positive control of the staining has been carried out **(b)**. For this, before the staining step, MDA-MB-231 spheroids were exposed with or without DFO (deferoxamine) at 250 µM, a well-known hypoxia inducer molecule. Staining was realised with ROS-ID orange reagent as previously described and hypoxia level was quantified by acquired fluorescent signal intensity with Gen5 software (BioTek®). Mean fluorescent intensity was of 36.2 ± 3.2×10^3 UF for DFO treated spheroids and significantly higher than 28.8 ± 4.1×10^3 UF ($p=0.009$, 2-sided t test) obtained for control spheroids. This experiment demonstrated that the ROS-ID orange reagent associated with our detection and quantification methodology is able to detect and quantify changes in global spheroid hypoxia level, induced by DFO.
